# Smartphone Addiction and Cybercrime Victimization in the Context of Lifestyles Routine Activities and Self-Control Theories: The User’s Dual Vulnerability Model of Cybercrime Victimization

**DOI:** 10.3390/ijerph18073763

**Published:** 2021-04-04

**Authors:** Juan Herrero, Andrea Torres, Pep Vivas, Antonio Hidalgo, Francisco J. Rodríguez, Alberto Urueña

**Affiliations:** 1 Department of Psychology, Universidad de Oviedo, 33000 Oviedo, Spain; olaizola@uniovi.es (J.H.); grupomito@hotmail.com (A.T.); gallego@uniovi.es (F.J.R.); 2 Department of Psychology, Universitat Oberta de Catalunya (UOC), Rambla del Poblenou, 156, 08018 Barcelona, Spain; pepvivasielias@gmail.com; 3 Departamento de Ingeniería de Organización, Administración de Empresas y Estadística ETSI Industriales José Gutiérrez Abascal, Universidad Politécnica de Madrid (UPM), 2. 28006-Madrid, Spain; antonio.hidalgo@upm.es

**Keywords:** cybercrime victimization, lifestyle-routine activities theory, self-control theory, smartphone addiction, national sample, dual vulnerabilities model of cybercrime victimization

## Abstract

(1) Background: This paper combines lifestyle-routine activities (L-RAT) and self-control (SCT) theories along with the literature on smartphone addiction in a joint model that addresses the multiple vulnerabilities that make the smartphone user a potential victim of cybercrime. This model, which we call the dual vulnerability model of cybercrime victimization, was subjected to empirical testing on a nationally representative sample of smartphone users. (2) Methods: Data from 2837 participants from a nationally representative sample of Spanish smartphone users were modeled using Mplus causal modeling software. (3) Results: The results of the study confirm the predictions of L-RAT and SCT in explaining cybercrime victimization (higher cybercrime victimization under conditions of high exposure, proximity, and suitability, relative absence of capable guardian, and low self-control). A significant effect of smartphone addiction on cybercrime victimization was also observed above and beyond L-RAT and SCT predictors. (4) Conclusions: The potential victim of cybercrime presents a double vulnerability, on the one hand, those identified by criminological theories such as L-RAT and SCT, and on the other hand, those derived from the deregulated-addicted use of the Internet access device (smartphone in our work).

## 1. Introduction

One of the most fertile theoretical approaches to cybercrime victimization in recent years has been the lifestyle-routine activities theory (L-RAT). This theory comes from the combination of two theories with a strong situational emphasis: the routine activities theory (RAT) and the lifestyle-exposure theory (LET). Consistent with rational choice theory [1], RAT focuses on the characteristics of crime rather than on those of the actual offenders [2]. RAT argues that crime events are produced by the intersection in time and space of a motivated offender, an attractive target, and a lack of capable guardianship [2]. While some authors focus on how opportunity for crime is structured by large-scale shifts in routine daily activity [2], LET theorists explain the differential risk of victimization as a function of the variation in lifestyles that can potentially expose people to offenders [3,4]. Those most at risk of crime victimization are more likely to have lifestyles that consist of spending more time in public (especially at night), more time away from family, and greater proximity to high-risk groups (e.g., young men). The L-RAT proposes that the risk of crime victimization is a function of the extent to which individuals are exposed to motivated offenders, live in proximity to motivated offenders, exhibit target suitability, and lack capable guardianship. The L-RAT has been supplemented with Hirsch’s theory of self-control (SCT) [5,6]. This later addition had its origin in the need for researchers of L-RAT to explain why some individuals engage in routines or lifestyles that exposes them to increased risk of victimization. The impact of low self-control on crime victimization is likely to be indirect, in large part by encouraging poor lifestyle choices that make people most vulnerable to crime [7].

### Literature Review

The key concepts of L-RAT such as capable guardian, proximity to offenders, target suitability, or exposure have a particular meaning when applied to street crime. When moving to the world of the Internet, however, some important clarifications are necessary [8,9]. Firstly, the concept of capable guardian changes substantially when L-RAT is used to explain cyber-crime: on the Internet, the guardian is basically the user, who is able to monitor their movements through certain mechanisms. These mechanisms include active and passive security strategies [10]. Active strategies refer to the set of actions that users carry out to try to minimize security threats (e.g., accessing dangerous websites, installing unverified software, downloading files with an unknown sender, etc.). Passive strategies refer to the installation of security barriers on devices that seek to protect the user from external threats (e.g., antivirus software).

Secondly, L-RAT highlights the fact that exposure to offenders may be more likely in certain risk situations. For example, spending a lot of time outside the home, and especially at night. In cyberspace this concept also needs to be translated to reflect the fact that there is something in the person’s cyber lifestyle that makes them more likely to be victimized (because they are more proximal and more exposed to offenders) [11]. Thus, those who interact in certain forums or frequently visit certain websites may be more exposed to potential cyber-criminals.

Thirdly, target suitability represents the attractiveness that a person, place, or object has for a would-be offender. These are characteristics that indicate to the offender that his attempts will be successful. For example, an old woman alone on the street with a handful of money bills is a very suitable target from the offender’s point of view. Translated into the online world where cybercrime takes place, the suitability of the target provides the offender with information on the potential success of his or her offense. Individuals who readily provide personal information online, or who have personal information publicly available may be easier targets for cyber criminals who are trying to build a profile for the would-be victim.

Fourthly, an additional element that L-RAT and other related situational theories have included in their explanations is the tendency of individuals to become involved in the type of situations that precisely increase their potential as victims. On this point, L-RAT scholars have borrowed from self-control theory (SCT) [5] the idea that people with an inability to consider the consequences of impulsively seeking immediate gratification (low self-control) will put themselves at greater risk of being victimized. L-RAT theorists have adapted this intrapersonal tendency to their postulates, in terms of a fundamentally indirect relationship [10]. People with low self-control tend to be more victimized because they are more exposed, more attractive to criminals, and more often neglect security measures. As a result, people with low self-control are more likely to be victimized.

These elements (exposure, proximity, suitability, absence of capable guardian, and low levels of self-control) currently constitute one of the most widely used conceptual frameworks to explain cybercrime victimization (see [10] for an analysis). These studies, however, do not usually take into account the fact that the entire conceptual framework applied to cybercrime also relies on the device through which the cyber space is accessed and the general use that the user makes of that device. In this vein, deregulated use of the device is in itself a risk factor for cybercrime victimization, an aspect that has important consequences for the potential cybercrime victimization of the user.

Therefore, the use of the smartphone has special relevance since it is probably the preferred device for users to interact in cyberspace. Thus, deregulated or addictive use of the smartphone may be a central element of the user’s vulnerability, making them prone to cybercrime victimization. Recent literature on smartphone addiction has pointed to the personality of the users (low conscientiousness and low agreeableness) and their emotional health (i.e., high depressive symptomatology, negative affect, etc.) [11] as being at the core of the personal factors that can foster addiction, while the lack of social connectedness (e.g., low social support) is an important social factor that can nurture addiction [12,13]. These persistent personal and social elements of the addicted user might make him or her psychosocially vulnerable, and this vulnerability must also be taken into account as an antecedent that activates habits of use and security that make them, in turn, also vulnerable to cybercrime victimization. It has been found, for example, that the terminals of smartphone addicted users tend to be more damaged in terms of the greater number of malware attacks and their harmful nature for the terminal [14,15], which suggests a direct relationship between addiction and neglect of safety.

While it may be true that the dispositional factor of low self-control may lead users to interact impulsively and without properly gauging the consequences (leading to a greater likelihood of being victims of cybercrime), smartphone addiction can help uncover other types of user vulnerabilities that can also make them prone to cybercrime victimization beyond low self-control. Feeling depressed, or relatively socially isolated can lead users to interact on the wrong sites, with the wrong people, provide them with highly sensitive information or neglect their most basic protection when interacting in cyberspace. High levels of smartphone addiction will therefore affect virtual lifestyles, and thus, potential cybercrime victimization. Although it has been highlighted that low self-control is a key element of addiction (the lower the self-control, the higher the addiction), this relationship is far from universal. Some studies have pointed out, for example, that self-control can simultaneously show both negative and positive relationships with addiction [16]. Therefore, low levels of self-control and addiction to the smartphone cannot be considered equivalent, although they are related. Additionally, although low self-control may be an important antecedent to addictive behavior, it does not necessarily lead to it. The addictive behavior adds new components with a potential positive relationship with cybercrime victimization, as is the case of a high psychosocial vulnerability (poor emotional health and low social connectedness). For example, a user with high levels of addiction may neglect his or her safety very occasionally in order to meet a compelling emotional need because he or she feels lonely, or because he or she feels very depressed, even if that user consistently monitors his or her safety on a daily basis.

**Figure 1:**
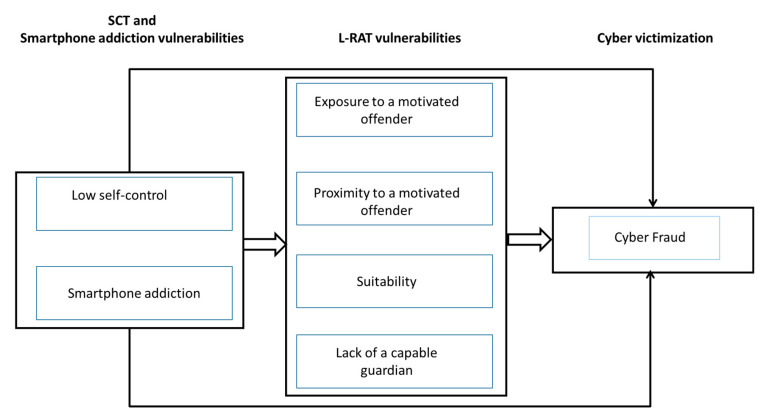
Vulnerabilities stemming from self-control theory (SCT), smartphone addiction and lifestyle-routine activities (L-RAT) and cyber fraud: the user’s dual vulnerability model of cybercrime victimization.

Although of undoubted scientific interest because of its ability to link two usually separate traditions of investigation (cybercrime victimization and smartphone addiction), this line of research has barely been explored. In this study, we analyzed the cybercrime victimization (cyber-fraud) of a national sample of smartphone users based on a dual user vulnerability model of cybercrime victimization: (a) the vulnerabilities proposed by L-RAT and SCT; and, (b) the vulnerabilities stemming from smartphone addiction (see Figure 1). The model in Figure 1 reflects a process of dual vulnerability of the user that might increase their potential cybercrime victimization. On the one hand, the model assumes the predictions of L-RAT theorists that certain situational aspects can lead to increased cybercrime victimization (exposure, proximity, suitability, and absence of a capable guardian). Additionally, the model includes dispositional aspects (low self-control) that may lead users to engage in such risky situations. The model also includes the way in which the internet access devices are used (the smartphone) and suggests that some of the vulnerabilities associated with deregulated use of the device could also affect the cybercrime victimization process.

According to our literature review, current studies on cybercrime victimization have paid little attention to the effect that smartphone addiction may have. Our research hypothesis is that smartphone addiction is an important element to consider in the explanation of cybercrime victimization along with the explanations proposed by L-RAT and SCT. The study had two objectives: (a) to contrast the effect of the vulnerabilities proposed by L-RAT and SCT on cybercrime victimization; and (b) to incorporate the vulnerabilities associated with the smartphone addiction in the prediction of cybercrime victimization.

## 2. Materials and Methods

### 2.1. Participants

We used data from the 2019 second semester Cybersecurity and Confidence in Spanish Households national survey (CCSHNS) conducted by the National Observatory of Telecommunications and Information Society of the Spanish Ministry of Industry for this study (see [17,18,19,20] for a detailed description). The data were collected between September and December 2019. Participants belonged to a representative sample of the Spanish population of Internet users over 15 years old, sampled within households. A total of 2837 respondents participated in the study.

The households were the primary sampling units and the secondary sampling units were the individuals within the households. The sample of households was representative in terms of autonomous communities, size of the locality, social class and number of people in the household. Secondly, Internet users over 15 years were identified and selected within households. A database with the study variables is available upon request to the authors.

### 2.2. Materials

#### 2.2.1. Dependent Variable

The dependent variable is cyber fraud. The survey evaluates cyber fraud with a dichotomous response question (0 = no, 1 = yes) addressed to participants: “Have you suffered any financial loss in the last 6 months due to possible cyber fraud?” About 10% of participants claimed to have suffered cyber fraud in the last 6 months (9.6%).

#### 2.2.2. Covariates

L-RAT covariates. (a) Exposure to motivated offenders. Exposure to motivated offenders was measured with five dichotomous response items (0 = no, 1 = yes) that evaluated the use during the last six months of various internet services with potential risk: file downloads, free content downloads, file sharing, visits to adult entertainment sites, and gambling services. Cronbach’s Alpha = 0.65; McDonald’s ω = 0.70. (b) Proximity to motivated offenders. To measure proximity to motivated criminals, we used seven items with a dichotomous response (0 = no, 1 = yes) that evaluated the extent to which users had suffered from cybercrime victimization attempts: requested user passwords, unsolicited service offers, unsolicited mail, suspicious or directly fraudulent job offers, offers from suspicious or false web pages, access to web pages that pretended to be banks, and having been registered for unsolicited services. Cronbach’s Alpha = 0.67; McDonald’s ω = 0.68. (c) Target suitability. Participants were asked whether they would provide personal information online via email or instant messaging and how much confidence they would have in doing so. Category responses were recoded so that higher scores reflected higher suitability (1 = No confidence to 5 = Much confidence). Since this was an attitudinal measure and not necessarily informative of the behaviors performed, we analyzed the relationship between the target suitability scores and the following item on social networks: “What kind of profile settings do you have applied regarding your visibility and security level?” (1 = My information can only be seen by some friends/contacts to 4 = My information can be seen by any user of the social network). Pearson’s zero order bivariate correlation (r = 0.08, *p* < 0.001, *n* = 2134) suggested that distrust of providing information is indeed related to a more restricted range of people allowed to access it on social networks. Thus, our measure of target suitability was informative of the tendency of the person to provide sensitive information on the Internet. (d) Capable guardianship. Participants responded about whether they used any of the following security measures on their device: unlocking pattern, automatic blocking, backup, and anti-virus (M = 2.60; SD = 1.15). Only a small percentage of participants reported not using any of the four security measures (*n* = 152, 5.4%).

SCT covariates. The CCSHNS measured self-control indirectly through two of the Big Five personality traits, agreeableness, and conscientiousness, which are thought to capture many elements (e.g., insensitivity to others and impulsivity) of self-control [5]. In this study we followed an approach consistent with using agreeableness and conscientiousness as indicators of self-control [21] (see [22] for an analysis). For this, we used the abbreviated version of the Big Five Inventory (BFI) [23], which measures with four items, agreeableness (2 items) (M = 3.36, SD = 0.79), and conscientiousness (2 items) (M = 3.74, SD = 0.76) (Cronbach’s α ≥ 0.65; McDonald’s ω ≥ 68). Recent research has found moderate to large correlations between agreeableness, conscientiousness, and self-control (r’s > 0.68, *p* < 0.001) [24]. Higher scores in agreeableness and conscientiousness were indicative of higher levels of self-control.

Smartphone addiction. The survey used eight items from the smartphone addiction symptoms scale (SAPS) [25] that are most conceptually equivalent to Young’s screening instrument of Internet addiction [26]. Scores from 1 to 5 (1—not true to 5—extremely true) were first dichotomized as 4 (true) or 5 (extremely true) = 1 and all remaining response categories = 0. Items were summed (M = 1.63, SD = 2.24) (Cronbach’s Alpha = 0.85; McDonald’s ω = 0.90).

#### 2.2.3. Control Variables

Sociodemographic. *Sex*. A total of 48.71% of participants were male (*n* = 1382) and 51.28% were female (*n* = 1455). *Age*. Age was measured in five age groups years: 15 to 24 years (9.2%), 25 to 34 years (17.9%), 35 to 44 years (28.9%), 45 to 54 years (24.6%), and more than 55 years (19.4%) (M = 3.27, SD = 1.22).

Social Desirability. The short form of the Marlowe-Crowne social desirability scale [27,28] was used to control for potential response bias. This short form has shown adequate psychometrical properties and consists of 10 true-false items from the original 33-item scale (1 = True, 2 = False). Negative items were reversed so that higher scale scores reflect greater levels of social desirability. Scores on all 10 items were summed and averaged (M = 1.55, SD = 0.19).

#### 2.2.4. Statistical Analyses

The model in Figure 1 was estimated by including sex, age, and social desirability as covariates (these relationships are not shown in Figure 1). For this, all the remaining variables of the model were predicted by the sociodemographic variables and social desirability. Covariations among sociodemographic variables and social desirability were freely estimated resulting in coefficients statistically controlled for the effect of the control variables [29]. All covariance among predictors were also freely estimated. The significance of indirect effects was evaluated with biased-corrected bootstrapped 95% confidence intervals [30]. It should be noted that the model in Figure 1 contains relationships that are evaluated by logistic regression (for the dependent variable “cyber fraud”) and linear regression (all other relationships in the model, except for the indicated covariations) techniques. The statistical package Mplus 8.2 [31] was used for the evaluation of the relationships presented in Figure 1.

## 3. Results

No evidence of multicollinearity was detected among the predictor variables (variance inflator factor (VIF) less than 2, and tolerance greater than 0.80). The results are presented in Table 1. As expected, all dimensions of L-RAT were directly and significantly related to cyber fraud. Users with greater exposure (OR = 1.12, 95% CI (1.01, 1.25)) and proximity (OR = 1.42, 95% CI (1.29, 1.57)) to cyber-criminals, more suitable as victims (OR = 1.34, 95% CI (1.21, 1.47)) and with fewer security measures (capable guardian) (OR = 0.88, 95% CI (0.80, 0.98)) showed a greater tendency to suffer cyber fraud.

Smartphone addiction presented a statistically significant association with all dimensions of L-RAT (not shown in Table 1): exposure to a motivated offender (β = 0.18, *p* < 0.001), proximity (β = 0.15, *p* < 0.001), target suitability (β = 0.15, *p* < 0.001), and capable guardian (β = 0.07, *p* < 0.01). Finally, both levels of self-control: agreeableness (OR = 0.80, 95% CI (0.67, 0.95)) and conscientiousness (OR = 0.67, 95% CI (0.57, 0.78)) and smartphone addiction (OR = 1.20, 95% CI (1.16, 1.26)) were directly related to cybercrime victimization.

The model in Figure 1 also incorporates the analysis of total and indirect effects (along with the direct ones already mentioned). The total effects indicate the absolute relationship of a variable with the dependent variable, while the direct and indirect effects suggest some paths by which this relationship could be explained.

Regarding the total effects of the model variables, higher rates of cyber fraud were observed in conditions of higher addiction to the smartphone (b = 0.25, *p* < 0.001), and lower self-control, especially for conscientiousness (b = −0.16, *p* < 0.001). In the case of the L-RAT variables, their total effects on cyber-fraud were equivalent to their direct effects on that variable. In the specific case of self-control and addiction, there were indirect effects, the breakdown of which is given below.

For the self-control variables, it was observed that the indirect effects of agreeableness and conscientiousness on cybercrime victimization were close to marginal (agreeableness, b = 0.04, 95% C.I. (0.01, 0.07); conscientiousness, b = −0.03, 95 % C.I. (−0.01, 0.05)). Specifically, agreeableness showed a marginal positive indirect effect while the effect of conscientiousness was not significant. Smartphone addiction also showed an indirect effect on cybercrime victimization (b = 0.05, 95% C.I. (0.03, 0.06)), through its effect on all dimensions of L-RAT already analyzed. Finally, the model explained 20% of the variability of the dependent variable.

In general, these results were indicative that both L-RAT and SCT make correct predictions about cybercrime victimization, while other complementary explanations (smartphone addiction) should also be taken into account.

Other results that also help to understand the analyzed model are the relationships observed among L-RAT and SCT components, and smartphone addiction. All L-RAT dimensions were significantly intercorrelated (r’s ≥ 0.08, *p*’s < 0.001) with two exceptions. Proximity and suitability were negatively correlated (r = −0.11, *p* < 0.001) and suitability and capable guardian were statistically unrelated (r = −0.01, *ns*.). SCT dimensions were significantly intercorrelated (r = 0.18, *p*< 0.001). Both agreeableness (r = −0.08, *p* < 0.001) and conscientiousness (r = −0.15, *p* < 0.001) were related to smartphone addiction, in line with other research [19].

## 4. Discussion

The empirical literature has shown some success in predicting cybercrime victimization from two theoretical approaches (RAT and LT), which researchers have integrated into L-RAT [10]. According to L-RAT, victims of cybercrime tend to show greater exposure and proximity to cybercriminals. In addition, these potential victims of cybercrime show a tendency to provide personal and sensitive information on the Internet (suitability), along with a lack of capable guardianship (negligence in cyber-security measures). A dispositional factor has also been identified that could explain the tendency of some users to interact under these potentially risky circumstances: low self-control (SCT). The combination of these three theoretical perspectives (RAT, LT, and SCT) has become one of the main areas of research in cybercrime [8,10]. However, these theoretical approaches tend to neglect an important factor; the relationship of the user with the device he or she interacts with on the Internet may largely condition this process. The smartphone is now the device of choice for most users, and addiction to this device has been linked to some vulnerabilities traditionally unattended by L-RAT and SCT researchers: showing poor emotional health, and low social connectedness, among the most important. In this study with 2837 smartphone users from a national representative sample, we incorporated smartphone addiction into the explanation of cybercrime victimization (cyber fraud) together with the main elements already highlighted by L-RAT and SCT.

The study had two objectives: (a) to contrast the effect of the vulnerabilities proposed by L-RAT and SCT on cybercrime victimization, and (b) to incorporate the vulnerabilities associated with the smartphone addiction to detect cybercrime victimization. All this was done with the help of statistical models that enabled the identification of the specific effect of each of these vulnerabilities on cybercrime victimization.

The achievement of the first objective confirmed the predictions of L-RAT and SCT almost completely. The greater the exposure, the proximity, and the suitability of the potential victim of cybercrime, and the relative absence of a capable guardian showed a clear predictive power for cybercrime victimization. Furthermore, these four elements of L-RAT were statistically associated with low self-control. As some scholars have predicted, the effect of low self-control on cybercrime victimization is eminently indirect in nature; low self-control enhances exposure, proximity and suitability while relaxing security measures (capable guardian) [8]. Our results do not seem to offer strong support for this hypothesis. On the contrary, most of the total effect of self-control on cybercrime victimization was direct; the greater the self-control in the dimensions of agreeableness and conscientiousness, the lesser the cyber-victimization. Although the original formulations of the SCT maintained that self-control was a one-dimensional disposition [5], this view has been challenged [32,33,34,35,36]. According to this view, the different dimensions of self-control might show a pattern of very diverse associations with the elements of L-RAT, so that indirect effects tend to be counterbalanced. According to our results, for example, the high self-control derived from high agreeableness is associated with a slight increase in proximity while the high self-control derived from high conscientiousness is related to low suitability. In this way, both positive and negative relationships were observed between some components of self-control and the main components of L-RAT.

The scientific literature in this field has pointed out that these two dimensions of personality reflect different forms of self-control. While agreeableness would relate to levels of insensitivity to others, conscientiousness would relate to levels of impulsivity, two different elements of self-control [21,22]. A comprehensive analysis of the differential effect of the various elements of self-control on cybercrime victimization is outside the scope of this study and future research should analyze these aspects in greater depth.

Perhaps the most unique contribution of this work is related to the second objective: addiction to the device used to access the Internet can lead to additional vulnerabilities to those considered by L-RAT and SCT that make users susceptible to cybercrime victimization. Our results confirmed that smartphone addiction is a very relevant explanatory factor and that its effect is independent of the effect found for the elements of L-RAT and SCT. Smartphone addiction was directly and positively related to higher cybercrime victimization rates (direct effect), but smartphone addiction was also positively related to greater exposure, greater proximity, and greater suitability. This indirect effect was substantial (around 20% of the total effect), which would give scientific support to the idea that in order to know in greater detail the tendency of users to put themselves in risk situations such as those indicated by L-RAT, research should also focus on the potential compulsive use of the device, Additionally, this effect would go beyond that found for low self-control. Regarding the unexpected positive relationship found between capable guardian and smartphone addiction, there is literature that has found a positive relationship between previous cybercrime victimization and capable guardian over time [37]. From this point of view, it is possible that users with higher addiction rates, and with a tendency to have been previously victimized by cybercrime, have developed some preventive security measures to avoid possible episodes of cybercrime victimization.

However, most of the effect found for smartphone addiction and cybercrime victimization was direct. It is interesting to note at this point that the observed influence of smartphone addiction on cybercrime victimization goes beyond low self-control, an aspect that has been consistently linked to addiction [38,39,40]. This fact leads us to believe that the psychosocial vulnerabilities associated with smartphone addiction play a very important role, as our results suggest. So regardless of the levels of self-control or the possible risk situations in which the user interacts, there is a very important differential element that turns addicted users into potential cyber victims. These distinctive elements include the poor emotional health and low social connectedness that characterize the addicted users [41].

According to this, even in users with high self-control who also monitor their interaction on the internet in an adequate way (they expose themselves little, they stay away from potential cybercriminals, they are reserved in the information they provide, or they take care of their cyber security) it is possible to find risks of cybercrime victimization if their use of the smartphone is deregulated to attend, probably, to more urgent needs of a psychosocial nature (poor emotional health and low social connectedness, for example). This opens up new avenues of research in the study of cybercrime victimization by incorporating psychosocial vulnerability into the equation.

### Strengths and Limitations

This paper presents some potential strengths. First, the participants belong to a nationally representative sample of Internet users, which is not common in this field, where convenience samples (e.g., university students) are the rule rather than the exception. This circumstance undoubtedly helps the potential generalization of the results. Secondly, variables from different conceptual perspectives (L-RAT, SCT, smartphone addiction) have been incorporated into the study, which has allowed us to obtain a more precise overview of cybercrime victimization. Related to the above, and thirdly, we believe that the attempt to combine various theoretical perspectives in the explanation of cybercrime victimization may help to broaden the development of this field of study.

Despite these strengths, the work also has potential limitations. First, a temporary panel design is needed to test the proposed model. Because variables were measured in the same time interval, the choice of the directionality of the relationships may seem arbitrary. In this sense, the fact that the observed statistical relationships were consistent with our conceptual predictions makes us confident in the plausibility of the model. Future work in this area should deepen the study of relationships over time. Secondly, the operationalization of the model may have had some influence on the results obtained. When L-RAT is applied to the study of cybercrime victimization, there is no consensus as to the operationalization of the main elements of these theories, that is, exposure, proximity, suitability, and capable guardian. Furthermore, our operationalization of self-control through agreeableness and conscientiousness may also have had some effect on the model. While SCT initially proposed that self-control was a disposition of a one-dimensional nature, there is currently some objection among researchers to assume this premise of the theory. In fact, some of the main self-control scales elaborated to reflect SCT have proved to be multidimensional, which supports our orientation [21], on which we have based this part of our study. Undoubtedly, new studies with a different perspective on operationalization of the variables are necessary to advance in this field.

## 5. Conclusions

Our study combines two research traditions with common, but traditionally separate interests. On the one hand, research that has focused primarily on the study of situational explanatory factors (L-RAT) of cybercrime victimization and later extended with the contributions of SCT, and on the other hand, research focused on the disruptive consequences of smartphone addiction, among which, those that have dealt with the relationship between deregulated smartphone use and cyber security stand out [18].

This study contributes to the empirical literature in this field in two ways. On the one hand, the results of the study give strong empirical support to the predictions of L-RAT. In the case of SCT, the support is partial since we have not been able to fully verify the indirect effect of SCT on cybercrime victimization through its effect on the components of L-RAT. Our results point to a significant direct effect of low self-control on cybercrime victimization. On the other hand, the results of the study allowed us to identify smartphone addiction as an important predictor of cybercrime victimization. This effect is important and occurs in two ways: it affects the L-RAT components (indirect effects), and directly influences cybercrime victimization (direct effect). This direct effect is particularly relevant as it suggests that regardless of whether users show a propensity for cybercrime victimization in the terms envisaged by L-RAT and SCT, users with greater addiction to the smartphone also show a greater tendency to cybercrime victimization.

## Figures and Tables

**Table 1:** Unstandardized and standardized parameter estimates of L-RAT, SCT, and smartphone addiction predictors of cyber fraud in a national representative sample of users (*n* = 2837).

	Cyber Fraud			
Variables	Direct Effects (Odds Ratio) ^1^	Direct Effects (Stand. Coefficient) ^1^	Indirect Effects	Total Effects
*L-RAT*				
Exposure	1.12 [1.01, 1.25]	0.06 [0.01, 0.10]	-	0.06 [0.01, 0.10]
Proximity	1.42 [1.29, 1.57]	0.17 [0.12, 21]	-	0.17 [0.12, 21]
Suitability	1.34 [1.21, 1.47]	0.15 [0.10, 0.20]	-	0.15 [0.10, 0.20]
Guardian	0.88 [0.80, 0.98]	−0.07 [−0.13, −0.01]	-	−0.07 [−0.13, −0.01]
*SCT*^2^ Agreeableness Conscientiousness	0.80 [0.67, 0.95] 0.67 [0.57, 0.78]	−0.08 [−0.14, −0.02] −0.15 [−0.21, −0.10]	0.01 [0.00, 0.02] −0.01 [−0.02, 0.00]	−0.07 [−0.13, −0.01] −0.16 [−0.22, −0.11]
*Smartphone addiction* ^2^	1.20 [1.16, 1.26]	0.20[0.16, 0.25]	0.05 [0.04, 0.07]	0.25 [0.22, 0.30]

^1^ Logistic regression odds ratio and 95% bias-corrected bootstrapped 95% Confidence Interval in brackets. ^2^ SCT variables and smartphone addiction coefficients on cyber-fraud are standardized logistic regression coefficients [95% bias-corrected bootstrapped interval in brackets]. To facilitate the comparison of the total effects, the standardized coefficients are presented. For L-RAT variables, the direct and total effects are identical, which allows us to find the total effect of these variables on cyber-fraud in terms of odds ratio.

## Data Availability

Data is available from the authors upon request.

## References

[1] Becker G.S. (2007). Crime and punishment: An economic approach. Economic Analysis of the Law.

[2] Cohen L.E., Felson M. (1979). Social change and crime rate trends: A routine activity approach. Am. Sociol. Rev..

[3] Cohen L.E., Kluegel J.R., Land K.C. (1981). Social inequality and predatory criminal victimization: An exposition and test of a formal theory. Am. Sociol. Rev..

[4] Hindelang M.J., Gottfredson M.R., Garofalo J. (1978). Victims of Personal Crime: An. Empirical Foundation for a Theory of Personal Victimization.

[5] Gottfredson M.R., Hirschi T. (1990). A General Theory of Crime.

[6] Schreck C.J. (1999). Criminal victimization and low self-control: An extension and test of a general theory of crime. Justice Q..

[7] Pratt T.C., Turanovic J.J., Fox K.A., Wright K.A. (2014). Self-control and victimization: A meta-analysis: Self-control and victimization. Criminology.

[8] Akdemir N., Lawless C.J. (2020). Exploring the human factor in cyber-enabled and cyber-dependent crime victimisation: A lifestyle routine activities approach. Internet Res..

[9] Whitty M.T. (2019). Predicting susceptibility to cyber-fraud victimhood. J. Financ Crime.

[10] Wilcox P., Cullen F.T. (2018). Situational opportunity theories of crime. Annu. Rev. Criminol..

[11] Busch P.A., McCarthy S. (2021). Antecedents and consequences of problematic smartphone use: A systematic literature review of an emerging research area. Comput. Human. Behav..

[12] Al-Kandari Y.Y., Al-Sejari M.M. (2020). Social isolation, social support and their relationship with smartphone addiction. Inf. Commun. Soc..

[13] Lapierre M.A., Zhao P. (2021). Smartphones and social support: Longitudinal associations between smartphone use and types of support. Soc. Sci. Comput. Rev..

[14] Williams M.L. (2016). Guardians upon high: An application of routine activities theory to online identity theft in Europe at the country and individual level. Br. J. Criminol..

[15] Reyns B.W., Henson B. (2016). The thief with a thousand faces and the victim with none: Identifying determinants for online identity theft victimization with routine activity theory. Int. J. Offender Ther. Comp. Criminol..

[16] Wilson S.J., Maclean R.R. (2013). Associations between self-control and dimensions of nicotine dependence: A preliminary report. Addict. Behav..

[17] Herrero J., Urueña A., Torres A., Hidalgo A. (2019). Socially connected but still isolated: Smartphone addiction decreases social support over time. Soc. Sci. Comput. Rev..

[18] Herrero J., Torres A., Vivas P., Urueña A. (2019). Smartphone addiction and social support: A three-year longitudinal study. Interv Psicosoc..

[19] Herrero J., Urueña A., Torres A., Hidalgo A. (2017). Smartphone addiction: Psychosocial correlates, risky attitudes, and smartphone harm. J. Risk Res..

[20] Urueña López A., Mateo F., Navío-Marco J., Martínez-Martínez J.M., Gómez-Sanchís J., Vila-Francés J., Serrano-López A. (2019). Analysis of computer user behavior, security incidents and fraud using Self-Organizing Maps. Comput. Secur..

[21] Van de Weijer S.G.A., Leukfeldt E.R. (2017). Big five personality traits of cybercrime victims. Cyberpsychol. Behav. Soc. Netw..

[22] Jensen-Campbell L.A., Knack J.M., Waldrip A.M., Campbell S.D. (2007). Do Big Five personality traits associated with self-control influence the regulation of anger and aggression?. J. Res. Pers..

[23] Rammstedt B., John O.P. (2007). Measuring personality in one minute or less: A 10-item short version of the Big Five Inventory in English and German. J. Res. Pers..

[24] Zhang G., Chen X., Xiao L., Li Y., Li B., Yan Z., Guo L., Rost D.H. (2019). The relationship between Big Five and self-control in boxers: A mediating model. Front. Psychol..

[25] Bian M., Leung L. (2015). Linking loneliness, shyness, smartphone addiction symptoms, and patterns of smartphone use to social capital. Soc. Sci. Comput. Rev..

[26] Young K.S. (1998). Internet addiction: The emergence of a new clinical disorder. Cyberpsychol. Behav..

[27] Strahan R., Gerbasi K.C. (1972). Short, homogeneous versions of the marlow-crowne social desirability scale. J. Clin. Psychol..

[28] Crowne D.P., Marlowe D. (1960). A new scale of social desirability independent of psychopathology. J. Consult. Psychol..

[29] Bentler P.M. (2006). EQS 6 Structural Equations Program Manual.

[30] Preacher K.J., Kelley K. (2011). Effect size measures for mediation models: Quantitative strategies for communicating indirect effects. Psychol. Methods.

[31] Muthén L.K., Muthén B.O. (1998). Mplus User’s Guide.

[32] Kim J., Hong H., Lee J., Hyun M.-H. (2017). Effects of time perspective and self-control on procrastination and Internet addiction. J. Behav. Addict..

[33] Burt C.H. (2020). Self-control and crime: Beyond gottfredson & hirschi’s theory. Annu. Rev. Criminol..

[34] Maloney P.W., Grawitch M.J., Barber L.K. (2012). The multi-factor structure of the brief self-control scale: Discriminant validity of restraint and impulsivity. J. Res. Pers..

[35] Perrone D., Sullivan C.J., Pratt T.C., Margaryan S. (2004). Parental efficacy, self-control, and delinquency: A test of a general theory of crime on a nationally representative sample of youth. Int. J. Offender Ther. Comp. Criminol..

[36] Piquero A.R., MacIntosh R., Hickman M. (2000). Does self-control affect survey response? Applying exploratory, confirmatory, and item response theory analysis to Grasmick et al.’s self-control scale. Criminology.

[37] Guerra C., Ingram J.R. (2020). Assessing the relationship between lifestyle routine activities theory and online victimization using panel data. Deviant. Behav..

[38] Copersino M.L. (2017). Cognitive mechanisms and therapeutic targets of addiction. Curr. Opin. Behav. Sci..

[39] Nigg J.T. (2017). Annual Research Review: On the relations among self-regulation, self-control, executive functioning, effortful control, cognitive control, impulsivity, risk-taking, and inhibition for developmental psychopathology. J. Child. Psychol. Psychiatry.

[40] Stautz K., Zupan Z., Field M., Marteau T.M. (2018). Does self-control modify the impact of interventions to change alcohol, tobacco, and food consumption? A systematic review. Health Psychol. Rev..

[41] Herrero J., Torres A., Vivas P., Arenas A., Urueña A. (2021). Examining the empirical links between digital social pressure, personality, psychological distress, social support, users’ residential living conditions, and smartphone addiction. Soc. Sci. Comput. Rev..

